# Grape seed proanthocyanidins inhibit the invasive potential of head and neck cutaneous squamous cell carcinoma cells by targeting EGFR expression and epithelial-to-mesenchymal transition

**DOI:** 10.1186/1472-6882-11-134

**Published:** 2011-12-21

**Authors:** Qian Sun, Ram Prasad, Eben Rosenthal, Santosh K Katiyar

**Affiliations:** 1 Department of Dermatology, University of Alabama at Birmingham, Birmingham, AL, USA; 2 Department of Surgery-Otolaryngology, University of Alabama at Birmingham, Birmingham, AL, USA; 3 Comprehensive Cancer Center, University of Alabama at Birmingham, Birmingham, AL, USA; 4 Nutrition Obesity Research Center, University of Alabama at Birmingham, Birmingham, AL, USA; 5 Birmingham Veterans Affairs Medical Center, Birmingham, AL, 35294, USA

## Abstract

**Background:**

Head and neck squamous cell carcinoma (HNSCC) is responsible for over 20,000 deaths every year in United States. Most of the deaths are due, in large part, to its propensity to metastasize. We have examined the effect of bioactive component grape seed proanthocyanidins (GSPs) on human cutaneous HNSCC cell invasion and the molecular mechanisms underlying these effects using SCC13 cell line as an *in vitro *model.

**Methods:**

The therapeutic effects of GSPs on cancer cell invasion were studied using Boyden chamber and wound healing assays. The effects of GSPs on the levels of various proteins related with cancer cell invasion were determined using western blot analysis.

**Results:**

Using *in vitro *cell invasion assays, we observed that treatment of SCC13 cells with GSPs resulted in a concentration-dependent inhibition of cell invasion of these cells, which was associated with a reduction in the levels of epidermal growth factor receptor (EGFR). Treatment of cells with gefitinib and erlotinib, inhibitors of EGFR, or transient transfection of SCC13 cells with EGFR small interfering RNA, also inhibited invasion of these cells. The inhibition of cell invasion by GSPs was associated with the inhibition of the phosphorylation of ERK1/2, a member of mitogen-activated protein kinase family. Treatment of cells with UO126, an inhibitor of MEK, also inhibited the invasion potential of SCC13 cells. Additionally, inhibition of human cutaneous HNSCC cell invasion by GSPs was associated with reversal of epithelial-to-mesenchymal transition (EMT) process, which resulted in an increase in the levels of epithelial biomarker (E-cadherin) while loss of mesenchymal biomarkers (vimentin, fibronectin and N-cadherin) in cells. Similar effect on EMT biomarkers was also observed when cells were treated with erlotinib.

**Conclusion:**

The results obtained from this study indicate that grape seed proanthocyanidins have the ability to inhibit the invasion of human cutaneous HNSCC cells by targeting the EGFR expression and reversing the process of epithelial-to-mesenchymal transition. These data suggest that GSPs can be developed as a complementary and alternative medicine for the prevention of invasion/metastasis of HNSCC cells.

## Background

Head and neck squamous cell carcinoma (HNSCC) affects more than 40,000 people in the United States annually and is responsible for over 20,000 deaths every year [1,2]. HNSCC often generates from critical organs including the oral cavity, larynx, pharynx, and tongue that play indispensable roles in increased mortality rate [1]. Head and neck cutaneous SCC is also very common. Advances in surgical and medical therapies for HNSCC have only modestly improved the mortality rate, which has remained at 50% for the last three decades [3-6]. It has been demonstrated that epidermal growth factor receptor (EGFR), one of the ErbB family of receptors, which is overexpressed in over 90% of HNSCC tumors, is a marker of poor prognosis in patients with HNSCC [7-9]. Mortality rate due to HNSCC is closely associated with its potent capacity to metastasize distantly. Therefore, an approach that decreases the metastatic ability of HNSCC cells may facilitate the development of an effective strategy for its treatment and/or prevention.

Naturally occurring agents, particularly bioactive dietary phytochemicals, may serve as appropriate candidates for the prevention or therapy of HNSCC metastasis. If these phytochemicals are safe and devoid of toxicities, these can be considered for the prevention of cancer cell invasion, migration or metastasis and thus can be utilized as complementary and alternative medicine and/or as adjuvant therapy for conventional cytotoxic therapies. Grape seed proanthocyanidins (GSPs) are such promising bioactive phytochemicals that have shown anti-carcinogenic effects in some tumor models and exhibit no apparent toxicity *in vivo *animal models [10-12]. GSPs contain primarily proanthocyanidins (89%), which constitute dimers, trimers, tetramers, and oligomers of monomeric catechins and/or (-)-epicatechins, as described previously [11]. Although GSPs have been shown to have anti-tumor effects [10], their chemotherapeutic effects on the invasive potential of HNSCC cells have not been explored.

In the current study, we assessed the chemotherapeutic effects of GSPs on the invasion potential of human head and neck cutaneous squamous cell carcinoma cells, as the invasion of cancer cells is a major event in the metastatic cascade. The invasion potential of cutaneous SCC cells was also compared with the invasion potential of human epidermoid carcinoma cells which were not found on head and neck sub-sites. For this purpose, two cutaneous SCC cells lines were selected: one is SCC13 which was generated from the squamous cell carcinoma of the facial (head) skin. Second cell line is A431 which is well known human epidermoid carcinoma cell line and is not related with head and neck sub-sites. In this study, we characterized the role of EGFR on the migration of head and neck cutaneous SCC cells and ascertained whether GSPs have any suppressive effects on the invasion of these cells and whether EGFR is involved in this process. Epithelial-to-mesenchymal transition (EMT), the process whereby epithelial cells transform into mesenchymal cells, has been shown to be relevant for cancer and cancer cell metastasis. During EMT, cancer cells lose expression of proteins that promote cell-cell contact such as E-cadherin and acquire mesenchymal markers such as vimentin, fibronectin and N-cadherin, which promote tumor progression, cell invasion and metastasis [13,14]. The EMT has also been associated with higher expression levels of EGFR and EGFR-mediated signaling, therefore we have also checked whether inhibition of EGFR expression by GSPs in head and neck cutaneous SCC cells is associated with reversal of EMT and that leads to inhibitory effect on cell invasion of head and neck cutaneous SCC cells. Here, we present evidence that GSPs inhibit the invasive potential or migratory behavior of head and neck cutaneous squamous cell carcinoma cells through inhibition or reversal of EMT and that GSPs do so through a process that involves the reduction in EGFR expression level.

## Methods

### GSPs, source and composition

GSPs were received from Kikkoman Biochemifa Company, Japan (no financial conflict of interest). Quality control of GSPs is maintained by the company on lot-to-lot basis. GSPs contain approximately 89% proanthocyanidins, with dimers (6.6%), trimers (5.0%), tetramers (2.9%) and oligomers (74.8%), as described earlier [10-12]. Based on vendor's information and analysis this product is stable for at least two years when refrigerated at 4°C. We have particularly selected this product as a source of proanthocyanidins because it is commercially available in purified form, and their composition is known and maintained on lot-to-lot basis by the commercial vendor. Some proanthocyanidins products are also commercially available in the market and their compositions are more or less comparable with the product used in this study.

### Cell lines and cell culture conditions

Human epidermoid carcinoma cells (A431) and human head and neck cutaneous SCC cells SCC13 were obtained from the American Type Culture Collection (Manassas, VA) and normal human epidermal keratinocytes (NHEK) were obtained from Cell Culture Core Facility of Skin Diseases Research Centre at the University of Alabama at Birmingham, AL. The cells were cultured as monolayers in DMEM supplemented with 10% heat inactivated fetal bovine serum, 100 μg/ml penicillin-streptomycin (Invitrogen, Carlsbad, CA), and kept in a humidified atmosphere of 5% CO_2 _at 37°C. The NHEK were cultured in keratinocyte growth medium supplemented with 5 ng/ml human recombinant epidermal growth factor and 0.05 mg/ml bovine pituitary extract (Gibco/Invitrogen, Carlsbad, CA) and maintained in an incubator under the identical conditions. Cells were seeded at a density of 1 × 10^6 ^cells per petri dish and allowed to attach for 24 h before treatment with GSPs or other treatment agents. The sub-confluent cells were treated with either various concentrations of GSPs or other agents such as gefitinib or erlotinib. The GSPs, erlotinib or gefitinib were dissolved in a small amount of dimethylsulfoxide (DMSO), which was added to the complete cell culture medium. The maximum concentration of DMSO in media was 0.1% (v/v). Cells treated with DMSO only served as a vehicle control. To determine the effect of GSPs on epidermal growth factor (EGF)-mediated effects, GSPs were added in cell culture medium at least 30 minutes before the treatment of the cells with EGF.

### Antibodies, chemicals and reagents

Boyden Chambers and polycarbonate membranes (8 μm pore size) for cell invasion assays were obtained from Neuroprobe, Inc. (Gaithersburg, MD). The antibodies specific to N-cadherin, fibronectin, EGF, EGFR, ERK1/2 and β-actin were obtained from Santa Cruz Biotechnology (Santa Cruz, CA), while antibodies for vimentin and E-cadherin were purchased from Cell Signaling Technology (Beverly, MA). The appropriate secondary antibodies conjugated with horseradish peroxidase were obtained from Invitrogen (Carlsband, CA).

### Cell invasion assay

The invasion capacity of SCC cells was determined *in vitro *using Boyden Chambers (Gaithersburg, MD). In this assay, two chambers were separated with matrigel coated Millipore membranes (6.5 mm diameter filters, 8 μM pore size), as detailed previously [15,16]. Briefly, cancer cells (1.5 × 10^4 ^cells/100 μl serum-reduced medium) were placed in the upper chamber of Boyden chambers, test agents were added alone, or in combination, to the upper chamber (200 μl), and the lower chamber contained the medium alone (150 μl). Chambers were assembled and kept in an incubator for desired time points. After incubation, cells from the upper surface of Millipore membranes were removed with gentle swabbing and the migratory cells on the lower surface of membranes were fixed and stained with crystal violet. The membranes were examined microscopically and cellular invasion was determined by counting the number of cells on membranes in at least 4-5 randomly selected fields using an Olympus BX41 microscope. Representative photomicrographs were obtained using a Qcolor5 digital camera system fitted to an Olympus BX41 microscope. Each cell invasion experiment was repeated at least three times.

### Scratch assay or wound healing assay

Scratch assay was performed to detect the cell migration ability of SCC13 cells, as detailed previously [15]. Briefly, SCC13 cells were grown to full confluency in six-well plates and incubated overnight in starvation medium, which contained only 0.5% FBS in DMEM cell culture medium. Cell monolayers were wounded with a sterile 100 μl pipette tip, washed with starvation medium to remove detached cells from the plates. Cells were left either untreated or treated with selected concentrations of GSPs in full medium and kept in a cell culture incubator for 48 h. After 48 h, medium was replaced with phosphate-buffered saline (PBS) buffer, the wound gap was examined and cells were photographed using an Olympus BX41 microscope fitted with digital camera.

### Western blot analysis

Following treatment of cells for the indicated time periods with or without the treatment of GSPs or any other agent, the cells were harvested, washed with cold PBS and lysed with ice-cold lysis buffer supplemented with protease inhibitors, as detailed previously [15,16]. Equal amounts of proteins (50 μg) were resolved on 10% Tris-Glycine gels and transferred onto a nitrocellulose membrane. After blocking the non-specific binding sites, the membrane was incubated with the primary antibody at 4°C overnight. The membrane was then incubated with the appropriate peroxidase-conjugated secondary antibody and the protein bands were visualized using the enhanced chemiluminescence reagents. The equal loading of protein samples on the gel was verified after re-probing the membrane with anti-β actin antibody.

### Statistical analysis

For cell invasion assays, the control and GSPs, gefitinib or erlotinib treatment groups or combined-treatment groups separately were compared using one-way analysis of variance (ANOVA) followed by *post hoc *Dunn's test using GraphPad Prism version 4.00 for Windows, GraphPad Software, San Diego, California, USA, http://www.graphpad.com. All quantitative data for cell migration are shown as the mean number of migrating cells ± SD/microscopic field, n = 3. In each case *P *< 0.05 was considered statistically significant.

## Results

### The invasive potential of head and neck cutaneous SCC13 cells was greater than A431 cells

**Figure 1:**
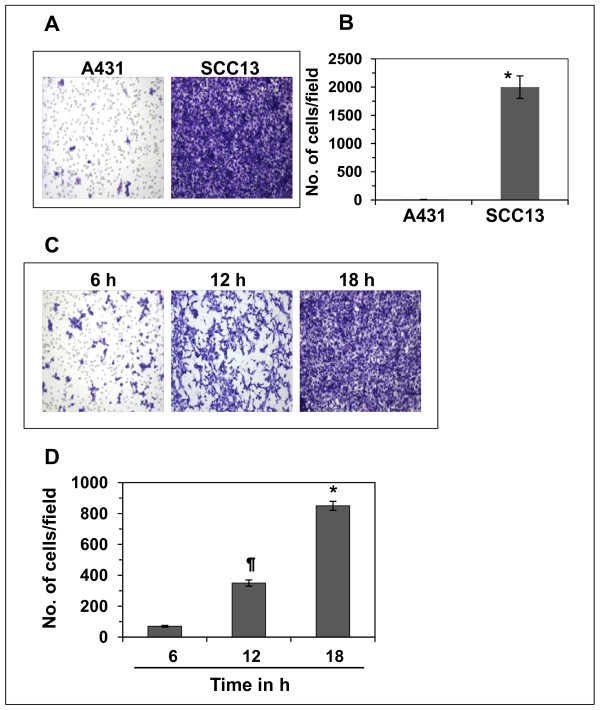
**Comparative cell invasion ability of human cutaneous squamous cell carcinoma cells**. **(A) **Invasive ability of human epidermoid carcinoma cells (A431) and head and neck cutaneous squamous cell carcinoma cells (SCC13) were compared. Equal numbers of cells were subjected to cell invasion using standard Boyden chamber assay. Twenty-four h later, invasive cells were detected on the membrane after staining with crystal violet. **(B) **The number of invasive cells were counted and the results expressed as the mean number of invasive cells ± SD/microscopic field (n = 3). Significantly higher *versus *A431 cells, *P < 0.0001. **(C) **Time-dependent cell invasion potential of human head and neck cutaneous SCC cells. **(D) **The invasive cells were counted and the results expressed as the mean number of invasive cells ± SD/microscopic field. Significant higher invasion of cells *versus *6 h time point, *P < 0.001; ^¶^*P *< 0.01.

First, we checked the invasive potential of head and neck cutaneous SCC13 cells and compared it with that of human epidermoid carcinoma cell line A431, which are not head and neck cancer cells, under identical experimental conditions. As shown in Figure 1A and 1B, the cell invasion ability of SCC13 cells was significantly higher (*P *< 0.0001) than A431 cells. The number of invasive SCC13 cells was 2000 ± 205 cells/microscopic field while the invasion of A431 cells was 12 ± 2 cells/microscopic field. These data indicate that cutaneous head and neck SCC cells are strongly aggressive in terms of their invasive potential than A431 cells which are not from the head and neck sites. Under identical conditions, the invasion potential of normal human epidermal keratinocytes was not observed (data not shown).

As SCC13 cells were highly invasive in nature, we examined the invasion ability of SCC13 cells at the early time points. As shown in Figure 1C, we could see the invasion of SCC13 cells as early as 6 h after the start of their incubation. The migration of SCC13 cells was time dependent. At 6 h time point, it was 70 ± 6; 12 h, 350 ± 20; and at 18 h, 850 ± 29 cells/microscopic field, as summarized in Figure 1D. After these preliminary observations, we selected 12 h time point for SCC13 cells for further studies on the invasive potential of this cell line and to examine the inhibitory effect of GSPs on its cell migration ability. Also, as the migrating capacity of A431 cells was extremely lower than SCC13 cells, we have selected only SCC13 cell line for further mechanistic studies.

### GSPs inhibit invasive potential of head and neck cutaneous SCC cells: Boyden chamber assay

**Figure 2:**
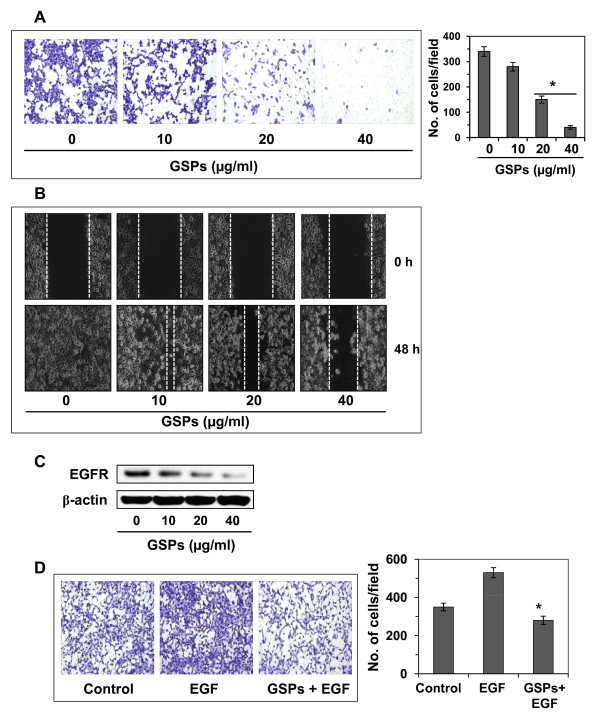
**Effect of GSPs on cutaneous HNSCC cell invasion**. **(A) **Treatment of cutaneous HNSCC cells (SCC13) with GSPs for 12 h inhibit invasion of cells in a concentration-dependent manner compared to non-GSPs-treated control cells. The invasive cells were counted and the results expressed as the mean number of invasive cells ± SD/microscopic field (right panel). Significant inhibition *versus *non-GSPs-treated control, *P < 0.001. **(B) **Wound healing assay was performed to assess the effect of GSPs on the migration of SCC13 cells. Incubation of SCC13 cells with GSPs for 48 h inhibits migration of cells in a concentration-dependent manner compared to non-GSPs-treated control cells. Broken white line indicates the gap without the presence of cells. Representative pictures are shown from three separate experiments under identical conditions. **(C) **Dose-dependent effect of GSPs on the EGFR expression of SCC13 cells. The levels of EGFR were determined in cell lysates using western blot analysis. **(D) **GSPs inhibit EGF-induced invasion of SCC13 cells. Treatment of SCC13 cells with EGF results in enhancement of cell invasion, and treatment of GSPs inhibit EGF-induced invasion of SCC13 cells. The data on cell invasion in each treatment group is summarized and the results expressed as the mean number of invasive cells ± SD/microscopic field (right panel). Significant inhibition *versus *EGF alone-treated group, *P < 0.001.

We determined whether treatment of SCC13 human head and neck cutaneous SCC cells with GSPs inhibited their invasiveness using Boyden chamber cell invasion assays. First, screening experiments were performed to determine the effects of lower (non-death inducing) concentrations of GSPs (μg/ml). As shown in Figure 2A, relative to untreated control cells, treatment of cells with GSPs at concentrations of 0, 10, 20 and 40 μg/ml reduced the invasive potential of SCC13 cells in a concentration-dependent manner. The density of the invasive cells on the membrane after staining with crystal violet is shown in Figure 2A, and the numbers of invasive cells/microscopic field are summarized in Figure 2A (right panel). The cell invasion was inhibited by18-85% (*P *< 0.01-0.001) in SCC13 cells in a concentration-dependent manner after treatment with GSPs for 12 h. To verify that the inhibition of invasion of SCC13 cells by GSPs was a direct effect on invasion ability, and that was not due to a reduction in cell viability/cell death, a trypan blue and/or MTT assays were performed [15] using cells that were treated identically to those used in the invasion assays. Treatment of SCC13 cells with various concentrations of GSPs (0, 10, 20 and 40 μg/ml) for 12 h had no significant effect on cell viability or cell death (data not shown).

### GSPs inhibit the migration of head and neck cutaneous SCC cells: Scratch or wound healing assay

As shown in Figure 2B, relative to untreated control cells, treatment of cells with various concentrations of GSPs (10, 20 and 40 μg/ml) reduced the migration capacity of SCC13 cells in a concentration-dependent manner after the treatment of cells for 48 h. The major part of gap or wounding space between cell layers after making a wound was occupied by the migrating SCC13 cells which were not treated with GSPs. However, the healing of the wound or the empty space between the cell layers was largely not occupied by the migrating cells treated with GSPs and this effect was dose-dependent. The gap or wounding space between the cells is highlighted by broken white lines (Figure 2B). These observations suggest that GSPs inhibited the migration of SCC13 cells. To further confirm that the inhibition of cancer cell migration by GSPs after 48 h was a direct effect on cell migration and not due to a reduction in cell viability, a trypan blue assay was performed using cells that were treated identically to those used in the migration assays. Treatment of SCC13 cells with various concentrations of GSPs (10, 20 and 40 μg/ml) for 48 h had no significant effect on cell viability or cell death (data not shown).

### The inhibitory effect of GSPs on invasive potential of SCC13 cells is associated with the reduction of EGFR expression

To determine whether the inhibitory effect of GSPs on the invasion of the SCC13 cells is associated with inhibition of EGFR expression, we determined the levels of EGFR in lysates of cells from the various treatment groups using western blot analysis. As shown in Figure 2C, treatment of SCC13 cells with GSPs for 12 h reduced the levels of EGFR expression in a concentration-dependent manner as compared to the expression in non-GSPs-treated controls. These results suggest that GSPs-induced reduction in EGFR expression may be associated with an inhibitory effect of the GSPs on the cell invasion of these cells.

### EGF, a ligand of EGFR, enhances the invasion of SCC13 cells, and GSPs inhibit EGF-induced cell invasion

EGF is a well known ligand of EGFR and has been shown to stimulate the activity of EGFR; therefore, the head and neck cutaneous SCC13 cells were treated with EGF (10 ng/ml) for EGFR stimulation, and thereafter determined the effect of EGF on the invasion of SCC13 cells. As shown in Figure 2D, treatment of SCC13 cells with EGF for 12 h resulted in significantly enhanced cell invasion (*P *< 0.01) compared to non-EGF-treated control cells. To determine whether GSPs inhibit EGF-induced cell invasion in human head and neck cutaneous SCC13 cells, SCC13 cells were treated with EGF (10 ng/ml) with and without the treatment of GSPs for 12 h. We found that the treatment of SCC13 cells with GSPs (40 μg/ml) resulted in significant inhibition (*P *< 0.001) of EGF-induced invasion of SCC13 cells. A summary of the cell invasion data for the different treatment groups is shown in Figure 2D (right panel).

### Selective EGFR inhibitors, gefitinib and erlotinib, inhibit the invasion of SCC13 cells

**Figure 3:**
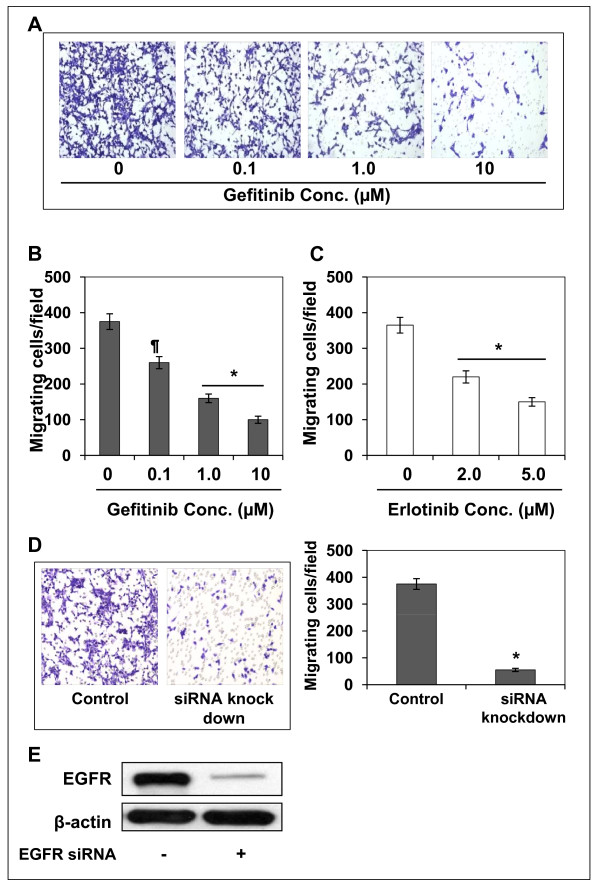
**Effect of gefitinib, an inhibitor of EGFR, on SCC13 cell invasion**. **(A) **Treatment of human head and neck cutaneous squamous cell carcinoma cells with gefitinib, a small molecule inhibitor of EGFR, for 12 h inhibits invasion of cells in a concentration-dependent manner. **(B) **The invasive cells were counted and the results expressed as the mean number of invasive cells ± SD per microscopic field. **(C) **Treatment of SCC13 cells with erlotinib, an inhibitor of EGFR, inhibited cell invasion. Significant reduction of cell invasion versus untreated control cells, ^¶^*P *< 0.05, *P < 0.001. **(D) **Transfection of cells with EGFR-siRNA significantly decreases cell invasion. SCC13 cells were transfected with EGFR-siRNA to knockdown EGFR expression (left panel). Significant reduction of cell invasion versus control siRNA-treated cells, *P < 0.001 (right panel). **(E) **Western blot analysis revealed that transfection of SCC13 cells with EGFR-siRNA resulted in marked reduction in the levels of EGFR in cells.

This experiment was performed to determine whether the inhibitory effect of GSPs on the cell invasion of head and neck cutaneous squamous cell carcinoma cells is mediated through its inhibitory effect on EGFR expression. For this purpose, SCC13 cells were subjected to the cell invasion assay after treatment with various concentrations of gefitinib (0, 0.1, 1.0 and 10.0 μM), a well known inhibitor of EGFR, for 12 h. As shown in Figure 3A, treatment of the cells with gefitinib resulted in a dose-dependent reduction in the cell invasion capacity of SCC13 cells as compared with non-gefitinib-treated controls (*P *< 0.05-0.001). These data suggested that the inhibition of constitutive levels of EGFR expression is associated with the inhibition of cell invasion of head and neck cutaneous squamous cell carcinoma cells. The resultant data on cell invasion/microscopic field at different doses of gefitinib are summarized in Figure 3B. Similar results were obtained when SCC13 cells were treated with another inhibitor of EGFR, erlotinib. Treatment of SCC13 cells with erlotinib for 12 h inhibited the invasion capacity of these cells, as shown by data summarized in Figure 3C.

### siRNA knock-down of EGFR reduces the invasion of SCC13 cells

We further verified the role of EGFR in cell invasion through siRNA knock-down of EGFR in the SCC13 cells using siRNA Transfection Reagent Kit (Santa Cruz Biotechnology, Inc., Santa Cruz, CA), and examined whether it would lead to the inhibition of the cell invasion in these cells. The data from cell invasion assay revealed that transfection of SCC13 cells with EGFR siRNA resulted in significant reduction of cell invasion (84%, *P *< 0.001) after 12 h as compared to the invasion of control siRNA-transfected SCC13 cells (Figure 3D, left and right panels). We also confirmed using western blot analysis that EGFR siRNA transfection of SCC13 cells resulted in marked reduction in the levels of EGFR protein (> 80%) in these cells (Figure 3E).

### GSPs inhibit the activation of ERK1/2 in SCC13 cells, and MEK inhibitor reduces the invasion potential of SCC13 cells

**Figure 4:**
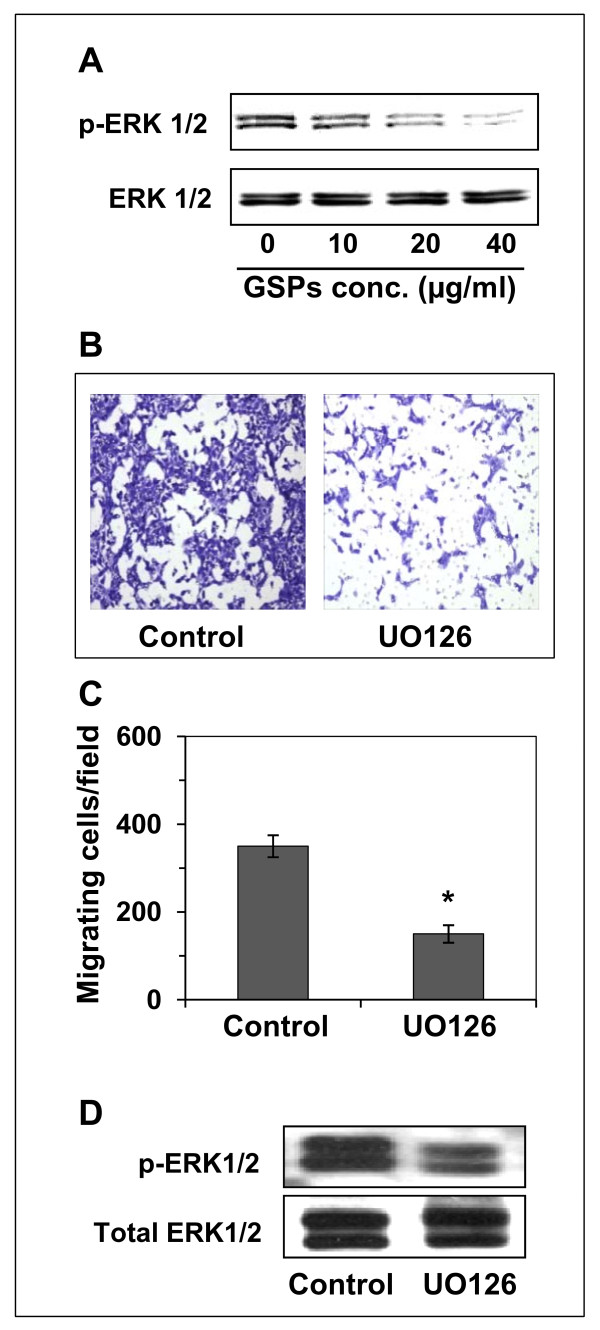
**GSPs inhibit SCC13 cell invasion by inhibiting activation of ERK1/2**. **(A) **GSPs inhibit the activation of ERK1/2 in SCC13 cells dose-dependently. **(B) **Treatment of SCC13 cells with MEK inhibitor (UO126, 80 μM) inhibits the invasion of cells compared to non-MEK inhibitor-treated control cells. **(C) **The data on cell invasion capacity are summarized and expressed as the mean number of invasive cells ± SD/microscopic field, n = 3. Significant difference versus control *P < 0.001. **(D) **The treatment of cells with MEK inhibitor also inhibits the activation of ERK1/2 in cells as determined by western blot analysis. Representative blot is shown from three separate experiments.

Mitogen-activated protein kinases (MAPK) are down-stream target of EGFR signaling, and have been implicated in cancer cell metastasis [17]. Therefore, we examined the effect of GSPs on activation of extracellular-signal regulated kinase (ERK1/2) in head and neck cutaneous SCC cells. Western blot analysis revealed that treatment of SCC13 cells with GSPs for 12 h inhibited the phosphorylation of ERK1/2 in a dose-dependent manner, as shown in Figure 4A. We further verified the role of activated ERK1/2 on SCC13 cell invasion by using the inhibitor of MEK (UO126). Cell invasion assay revealed that treatment of SCC13 cells with UO126 for 12 h significantly inhibited (*P *< 0.001) the invasion of cells (Figure 4B). A summary of data obtained from three independent experiments related with cell invasion is shown in Figure 4C. Additionally, western blot analysis revealed that the level of phosphorylated ERK1/2 was also decreased after the treatment of cells with MEK inhibitor UO126, as shown in Figure 4D.

### GSPs reverse epithelial-to-mesenchymal transition in SCC13 cells

**Figure 5:**
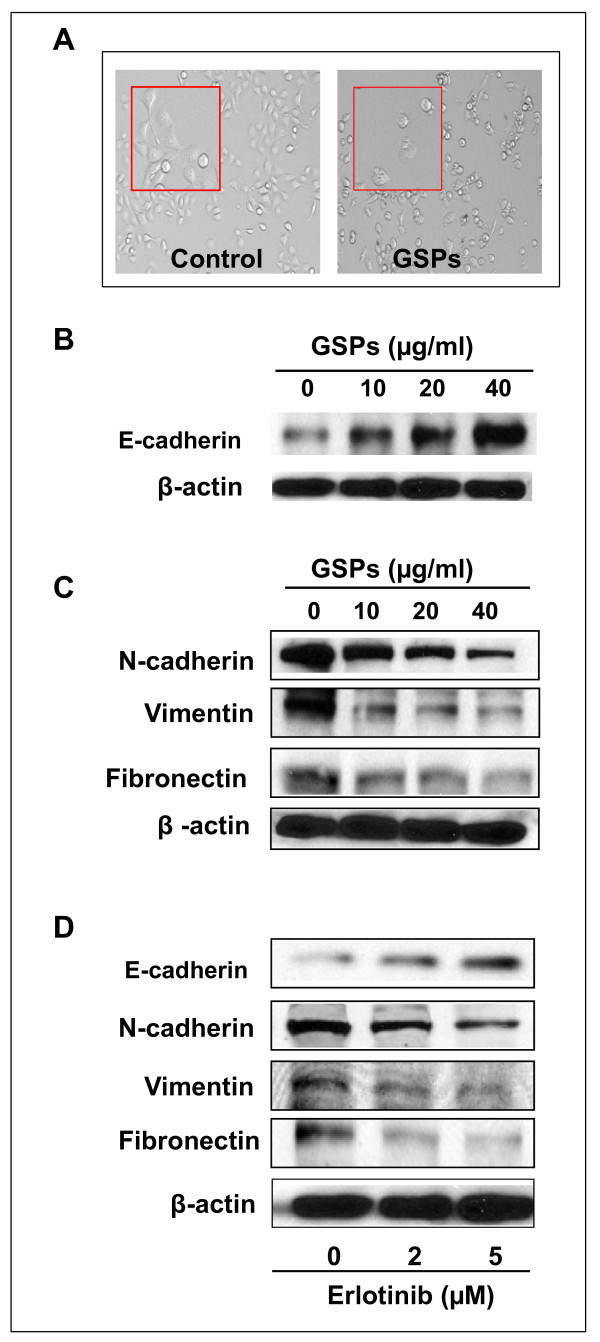
**Treatment of SCC13 cells with GSPs reverses epithelial-to-mesenchymal transition**. **(A) **Treatment of SCC13 cells with GSPs for 12 h resulted in loss of mesenchymal structure or morphology of cells. Cells lost their spindle shape morphology. Inset pictures showed magnified cell structure. **(B) **Treatment of cells with GSPs enhances the levels of E-cadherin, an epithelial biomarker, in the cells dose-dependently. **(C) **Treatment of cells with GSPs decreases the levels of mesenchymal biomarkers in SCC13 cells, such as N-cadherin, vimentin and fibronectin, in a dose-dependent manner. **(D) **Treatment of SCC13 cells with erlotinib, an inhibitor of EGFR, for 12 h decreases the levels of mesenchymal biomarkers, such as N-cadherin, vimentin and fibronectin.

Upregulation of EGFR and activation of downstream targets like ERK1/2 play a critical role in EMT [13,17], which in turn has been involved in cancer cell invasion and metastasis. To check if GSPs affect EMT in HNSCC cells, we examined if there is any change in SCC13 cells morphology after their treatment with low-dose GSPs under an inverted phase-contrast microscope (Figure 5A). For this purpose cells were treated with and without GSPs (20 μg/ml) for 12 h. As shown in Figure 5A, we found that culturing cells with GSPs for 12 h resulted in morphological changes of these cells from a spindle shaped or fibroblast-like shape to an epithelial-like shape. This change on cell morphology suggested that there was a transition of mesenchymal state to epithelial state under the influence of GSPs.

Next, we determined whether GSPs affect or reverse the biomarkers of EMT in head and neck cutaneous SCC cells and that is responsible for their inhibitory effect on the invasiveness of SCC13 cells. For this purpose, SCC13 cells were treated with GSPs for 12 h, and cell lysates were prepared for the western blot analyses of various epithelial and mesenchymal biomarkers. Western blot analyses revealed that GSPs increased the levels of E-cadherin, an epithelial biomarker, in SCC13 cells in a dose-dependent manner compared to untreated controls (Figure 5B). In contrast, the levels of mesenchymal biomarkers, such as N-cadherin, vimentin and fibronectin, were reduced in SCC13 cells after treatment with GSPs in a dose-dependent manner, as shown in Figure 5C. Similarly, treatment of SCC13 cells with erlotinib, an inhibitor of EGFR, for 12 h resulted in reduced expression of mesenchymal biomarkers, such as N-cadherin, vimentin and fibronectin, as evident by the western blot analysis (Figure 5D).

## Discussion

The metastasis of cancer cells is considered as a major cause of human death and mortality in any type of cancer. Treatment is difficult if cancer cells spread beyond the primary site of the tumor. Therefore, innovative strategies are required to be developed for the prevention of the invasive potential of cancer cells. In this study we found that head and neck cutaneous SCC cells are much more aggressive in terms of their invasion potential than other human skin cancer cells, such as A431 cells, which are well known human epidermoid carcinoma cells. Milliri et al [18] reported that the invasion potential of SCC-derived cells is dependent upon EGF stimulation, and this response to EGF does not occur in benign epidermal cells. Also, this response does not occur in A431 cells because these cells have sustained expression of the c-Jun deletion mutant, TAM67, which inhibits EGF-induced cytoskeletal rearrangements necessary for lamellipodia formation and cell rounding and ultimately cell motility and invasion.

The significant findings in the present study are that the treatment of head and neck cutaneous SCC cells with GSPs inhibits invasive potential of cells in a dose-dependent manner, and that is associated with the down-regulation of EGFR expression in cells. The head and neck cutaneous SCC13 cells over-express EGFR, and the inhibition of EGFR by GSPs contributes to the inhibition of cell invasion of these cells. This concept is supported by the evidence that treatment of the SCC13 cells with gefitinib or erlotinib, which are potent inhibitor of EGFR, resulted in a reduction of cell invasion. Similar effects were also noted when the SCC13 cells were transfected with EGFR-siRNA. Treatment of cells with EGF stimulates EGFR, and we observed that treatment of SCC13 cells with EGF enhances cell invasion ability, and that this EGF-induced cell invasion was blocked by the treatment of cells with GSPs. These observations support the evidence that inhibition of head and neck cutaneous squamous cell carcinoma cell invasion by GSPs is mediated through their inhibitory effects on EGFR expression. It has been reported that inhibitors of EGFR can prevent the growth and progression of HNSCC; however, long term use may also induce some form of toxicity [19,20]. This possibility is not expected with the use of GSPs as these are dietary components and toxicity has not been observed in animal models [11,12].

Proteins of MAPK family are a downstream target of EGFR, and have been shown to play a crucial role in cancer cell invasion. Our results show that inhibition of invasion of SCC13 cells by GSPs is associated with the inhibition of ERK1/2 phosphorylation. The inhibition of MEK with UO126, a MEK inhibitor, blocked the invasion capacity of SCC13 cells which is similar to the action of GSPs. These observations suggest a possible involvement of ERK1/2/MAPK pathway in inhibition of the invasion of cutaneous HNSCC cells. Activation of the proteins of MAPK family leads to the activation NF-κB which play an important role in multiple biological processes, including inflammation, cell proliferation and angiogenesis [21-23]. Importantly, NF-κB has been identified as an important regulator of EMT in several cancer cell types [21-24]. EMT has been implicated in invasion and metastasis of epithelial tumors. EMT can render tumor cells migratory and invasive through the involvement of all stages: invasion, intravasation and extravasation [13,14]. During the process of EMT, cells can change from an epithelial to a mesenchymal state. They lose their characteristic epithelial traits and instead gain properties of mesenchymal cells. This process is primarily coordinated by the disappearance or loss of epithelial biomarkers such as E-cadherin with the concomitant appearance or gain of mesenchymal markers such as vimentin, fibronectin and N-cadherin, etc. In the present study, GSPs treatment of SCC13 cells showed the suppression of mesenchymal biomarkers, such as vimentin, fibronectin and N-cadherin while restored the levels of epithelial biomarker such as, E-cadherin, in human cutaneous head and neck SCC cells which suggest that GSPs have the ability to reverse the EMT process in HNSCC cells. These information suggest that reversal of EMT in SCC13 cells by GSPs may also be one of the possible mechanisms through which GSPs reduce the invasiveness of cutaneous head and neck squamous cell carcinoma cells and that lead to inhibition of invasion of SCC13 cells in our system. A recent study showed that GSPs inhibit invasion of melanoma cancer cells and this inhibitory effect of GSPs on melanoma cell invasion was associated with their inhibitory effect on COX-2 overexpression and successive down-regulation of NF-κB and reversal of EMT process [25]. Similar to GSPs, other phytochemicals, such as berberine, have also been shown to inhibit the invasion potential of cancer cells. Berberine inhibits the invasion of melanoma cancer cells through its inhibitory effect on endogenous COX-2 overexpression and successive down-regulation of prostaglandin E2 and prostaglandin E2 receptors [16].

## Conclusion

**Figure 6:**
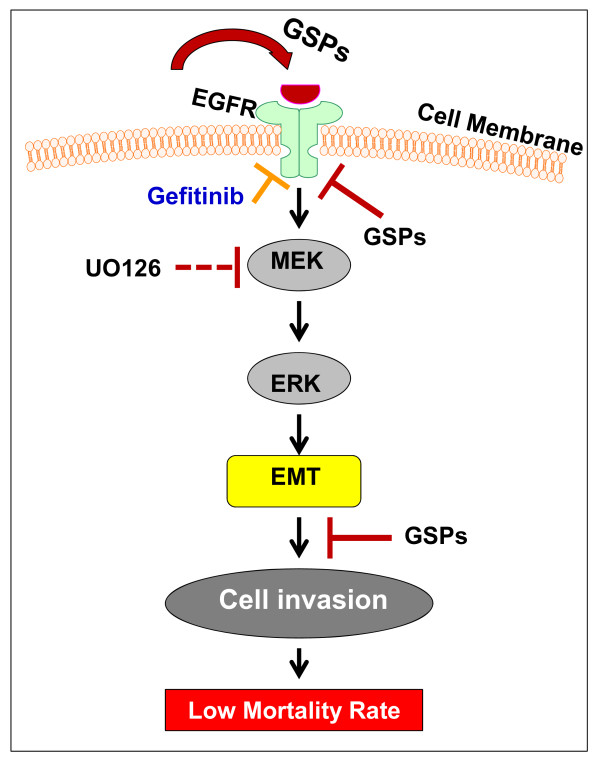
**Summary of GSPs action on the prevention of SCC13 cell invasion**. GSPs inhibit the expression levels of EGFR in human cutaneous head and neck squamous cell carcinoma cells, which plays a critical role in inactivation of ERK1/2 and reversal of epithelial-to-mesenchymal transition and that leads to inhibition of cell invasion potential.

The results from this study have identified for the first time that GSPs inhibit the invasiveness of human cutaneous HNSCC cells and that involves: (i) the inhibitory effect of GSPs on endogenous EGFR overexpression, (ii) the inhibitory effect of GSPs on the activation of the ERK1/2 proteins of MAPK family, and (iii) the reversal of EMT process, as summarized in Figure 6. More detailed studies are needed to develop GSPs as a pharmacologically safe agent either alone or in combination with other anti-metastatic drugs for the treatment of cutaneous head and neck SCCs in humans.

## List of abbreviations

EGF: epidermal growth factor; EGFR: epidermal growth factor receptor; EMT: epithelial-to-mesenchymal transition; ERK1/2: extracellular signal related kinases; GSPs: grape seed proanthocyanidins; HNSCC: head and neck squamous cell carcinoma; MAPK: mitogen-activated protein kinases; NF-κB: nuclear factor-kappaB; NHEK: normal human epidermal keratinocytes; SCC: squamous cell carcinoma.

## Competing interests

The authors declare that they have no competing interests.

## Authors' contributions

The work reported in this manuscript was done in collaboration with all authors. QS and RP have performed all experimental work, cell migrations assays, western blot analysis and statistical analysis of data. SKK is a principal investigator of the study, has designed the study, provided all supervision on daily basis, data analysis and write the final draft of the manuscript. ER was associated in data interpretation, discussion and study design, as well as final drafting of the manuscript. All authors read and approved the final manuscript.

## Pre-publication history

The pre-publication history for this paper can be accessed here:

http://www.biomedcentral.com/1472-6882/11/134/prepub
